# Risk factors and implications associated with ultrasound‐diagnosed nephrocalcinosis in cats with chronic kidney disease

**DOI:** 10.1111/jvim.17034

**Published:** 2024-03-04

**Authors:** Pak‐Kan Tang, Rebecca F. Geddes, Yu‐Mei Chang, Rosanne E. Jepson, Dirk Hendrik Nicolaas van den Broek, Nicola Lötter, Jonathan Elliott

**Affiliations:** ^1^ Department of Comparative Biomedical Sciences, Royal Veterinary College University of London London UK; ^2^ Department of Clinical Science and Services, Royal Veterinary College University of London London UK; ^3^ Research Support Office, Royal Veterinary College University of London London UK; ^4^ Department of Clinical Sciences, Faculty of Veterinary Medicine Utrecht University Utrecht The Netherlands

**Keywords:** CKD‐MBD, feline, hypercalcemia, mineralization, nephrolithiasis, radiology and diagnostic imaging, ultrasonography

## Abstract

**Background:**

Microscopic nephrocalcinosis is a common pathological feature of chronic kidney disease (CKD) in cats. Detection of macroscopic nephrocalcinosis using ultrasonography and its implications remain unexplored.

**Objectives:**

Identify risk factors associated with ultrasound‐diagnosed nephrocalcinosis and evaluate the influence of nephrocalcinosis on CKD progression.

**Animals:**

Thirty‐six euthyroid client‐owned cats with CKD.

**Methods:**

Prospective cohort study. Cats with CKD with and without ionized hypercalcemia were enrolled for renal ultrasonography. Cats were categorized according to the presence or absence of ultrasound‐diagnosed nephrocalcinosis. Binary logistic regression was performed to identify nephrocalcinosis risk factors. The influence of nephrocalcinosis on CKD progression was assessed using linear mixed models.

**Results:**

Ultrasound‐diagnosed nephrocalcinosis was evident in 61% of CKD cats overall, with increased prevalence (81%) in those with hypercalcemia. At enrollment, higher blood ionized calcium concentration (odds ratio [OR], 1.27 per 0.1 mg/dL; *P* = .01), plasma phosphate concentration (OR, 1.16 per 0.1 mg/dL; *P* = .05), plasma creatinine concentration (OR, 1.29 per 0.1 mg/dL; *P* = .02) and alanine aminotransferase activity (OR, 2.08 per 10 U/L; *P* = .04) were independent nephrocalcinosis risk factors. The rate of change in log‐transformed fibroblast growth factor‐23 differed significantly between groups (*P* = .04). Cats with CKD and nephrocalcinosis had increasing plasma creatinine concentrations (.03 ± .01 mg/dL/month; *P* = .04) and phosphate concentrations (.06 ± .02 mg/dL/month; *P* < .001) and decreasing body weight (.02 ± .01 kg/month; *P* < .001) over time.

**Conclusions and Clinical Importance:**

Nephrocalcinosis is prevalent in cats with CKD, especially in those with hypercalcemia. This pathological feature appears to be associated with CKD progression in cats.

**Abbreviations**
ALP: alkaline phosphatase
ALT: alanine aminotransferase
BCS: body condition score
CaPP: calcium phosphate product
CKD: chronic kidney disease
CKD‐MBD: chronic kidney disease‐mineral and bone disorder
CONSORT: Consolidated Standards of Reporting Trials
CPP: calciprotein particles
CPP‐1: primary calciprotein particles
CPP‐2: secondary calciprotein particles
CT: computed tomography
DICOM: Digital Imaging and Communications in Medicine
EDTA: ethylenediaminetetraacetic acid
ESRD: end‐stage renal disease
FGF23: fibroblast growth factor‐23
HCO_3_^−^
: bicarbonate
iCa: ionized calcium
IRIS: International Renal Interest Society
ln: natural logarithm
ln[FGF23]: log‐transformed fibroblast growth factor‐23
ln[PTH]: log‐transformed parathyroid hormone
MCS: muscle condition score
micro‐CT: microcomputed tomography
MPRD: moderately phosphate‐restricted diet
OR: odds ratio
PRD: phosphate‐restricted diet
PTH: parathyroid hormone
ROC: receiver operating characteristic
SBP: systolic blood pressure
SDMA: symmetric dimethylarginine
tCa: total calcium
tMg: total magnesium
TT4: total thyroxine
USG: urine specific gravity
UTI: urinary tract infection
*β*
: coefficient
Δ: delta

## 1 INTRODUCTION

Visualization of nephrocalcinosis without magnification is termed macroscopic nephrocalcinosis (referred to herein as nephrocalcinosis) and ultrasonography is the preferred imaging modality in humans.^1^, ^2^ Nephrocalcinosis is prevalent in human CKD patients, especially in dialysis patients with end‐stage renal disease (ESRD).^3^, ^4^ Total calcium (tCa) concentration is an independent risk factor for microscopic nephrocalcinosis in both cats and humans with CKD,^4^, ^5^ and its manifestation (both microscopic and macroscopic) previously has been associated with accelerated deterioration of renal function in studies of human patients,^6^, ^7^, ^8^, ^9^ but not all studies are in agreement and some found no relationship between renal calcification and renal function.^10^, ^11^, ^12^

Microscopic nephrocalcinosis has been documented previously in cats with azotemic CKD.^13^ In a histomorphometric study using necropsy samples, ≥50% of cats with International Renal Interest Society (IRIS) stage 2‐4 CKD had evidence of renal tubular mineralization.^13^ In a second retrospective study, up to 78% of CKD cats had evidence of nephrocalcinosis at necropsy.^5^ Despite the relatively high prevalence of microscopic nephrocalcinosis identified in CKD cats, our knowledge of its onset and progression are limited. Additionally, a recent study evaluated detection of nephrocalcinosis using ultrasonography, microcomputed tomography (micro‐CT) and histopathology, and found a strong positive correlation between macroscopic and microscopic nephrocalcinosis in cats with azotemic CKD.^14^ This finding suggested that ultrasonography can reliably detect nephrocalcinosis in cats.

Little is known about the implications of nephrocalcinosis for cats with CKD and whether intervention is required to ameliorate this potentially harmful pathological feature. We hypothesized that CKD cats with hypercalcemia would be at increased risk of developing nephrocalcinosis and that this pathological feature would be associated with CKD progression. Therefore, the aims of our prospective study were firstly, to assess the prevalence of nephrocalcinosis using ultrasonography in azotemic CKD cats with and without hypercalcemia; secondly, to explore the risk factors associated with nephrocalcinosis at the time of enrollment; and lastly, to determine the implications over time associated with nephrocalcinosis in these cats.

## 2 METHODS

### 2.1 Case selection

Azotemic CKD cats from 2 London‐based first‐opinion practices were prospectively recruited between March 2021 and July 2022. The study protocol was reviewed and approved by the Royal Veterinary College Ethics and Welfare Committee (URN20131258E). Azotemic CKD diagnosis was defined as a plasma creatinine concentration ≥2 mg/dL with concurrent urine specific gravity (USG) <1.035, or plasma creatinine concentration ≥2 mg/dL on 2 consecutive occasions without evidence of a pre‐renal cause. All owners of cats with a diagnosis of CKD initially were advised to feed a phosphate‐restricted diet (PRD; Feline Veterinary Diet Renal [dry and wet], Royal Canin SAS, Aimargues, France [dry] and Masterfoods, Bruck, Austria [wet]) with a phosphorus content of 0.7 to 1.1 g/Mcal and calcium‐to‐phosphorus ratio (Ca:P) of 1.3 to 2. Owners of cats that developed blood ionized (iCa) hypercalcemia (≥6 mg/dL [1.5 mmol/L] or ≥5.6 mg/dL [1.4 mmol/L] on 2 consecutive visits) were advised to feed a moderately phosphate‐restricted diet (MPRD; Feline Veterinary Early Renal [dry and wet], Royal Canin SAS, Aimargues, France) with a phosphorus content of 1.5 g/Mcal and Ca:P of 1.3. Cats that did not accept a PRD or MPRD were continued on their maintenance diets. Enrollment in this prospective study could occur at any time after a diagnosis of azotemic CKD, regardless of plasma creatinine concentration.

For inclusion in the study, measurements of iCa and plasma total calcium (tCa) concentrations were required on enrollment when baseline ultrasound scanning was performed. Based on these measurements, cats were classified into the following groups: Hypercalcemic group—iCa ≥5.6 mg/dL (1.4 mmol/L) on enrollment or ≥5.48 mg/dL (1.37 mmol/L) with historical measurements of either iCa ≥5.6 mg/dL (1.4 mmol/L) or tCa ≥11.8 mg/dL (2.95 mmol/L) during the previous 12 months; Normocalcemic group—iCa within the reference interval of 4.76‐5.48 mg/dL (1.19‐1.37 mmol/L)^15^ with all iCa measurements during the preceding 12 months being <5.48 mg/dL (1.37 mmol/L) and tCa <10.32 mg/dL (2.58 mmol/L).

The cut‐off for tCa was chosen to minimize the risk of prior undetected ionized hypercalcemia.^15^ Cats were excluded if they had suspected hyperthyroidism with plasma total thyroxine (TT4) concentration >40 nmol/L or were receiving medical management for hyperthyroidism, diabetes mellitus, recurrent urinary tract infection (UTI), or had evidence of other concurrent disease. Cats also were excluded if they were currently being fed a diet to manage urolithiasis, or were being treated with corticosteroids, furosemide, bisphosphonates or calcium‐based phosphate binders. Cats receiving amlodipine besylate for systemic hypertension were eligible for enrollment. Renal ultrasound scanning was performed on awake cats with owner informed consent. Cats that could not be scanned in the awake state were excluded.

### 2.2 Sample size and recruitment

A power calculation was performed assuming a 3‐fold difference in nephrocalcinosis prevalence between CKD cats with and without hypercalcemia^5^, ^13^ and indicated that 26 cats (13 in each group) were required to be able to reject the null hypothesis that there is no difference in nephrocalcinosis prevalence between groups, with 80% power and 5% type I error rate.

### 2.3 Clinicopathological data and study follow‐up

The schematic protocol of this 12‐month longitudinal study is illustrated in Supplementary Figure 1. Signalment of the cats enrolled, including sex, breed and age, was documented. At each visit, systolic blood pressure (SBP) measurements were obtained using a previously described Doppler method,^16^ followed by physical examination including body weight, body condition score (BCS, 9‐point scale) and muscle condition score (MCS, 4‐point scale).^17^ Indirect ophthalmoscopy was performed using a retinal camera (ClearView, Optibrand, Fort Collins, Colorado) in cats with average SBP >160 mm Hg. Systemic hypertension was defined as average SBP >160 mm Hg in conjunction with evidence of hypertensive retinopathy, or SBP >170 mm Hg on 2 consecutive occasions.

At the baseline visit, visits 2 (16‐20 weeks), 4 (32‐36 weeks) and 6 (48‐52 weeks), blood samples were collected by jugular venipuncture into heparinized, ethylenediaminetetraacetic acid (EDTA) and plain tubes, and urine was obtained by cystocentesis. Samples were stored at 4°C for <6 hours before centrifugation and separation. Biochemistry profiles were performed on heparinized plasma at an external laboratory (IDEXX laboratories, Wetherby, UK). Ionized calcium was quantified immediately on untreated whole blood or on electrolyte‐balanced heparinized whole blood within 5 minutes of venipuncture,^18^ using a point‐of‐care blood analyzer (*i‐STAT 1*, Abbott Point of Care Inc., Princeton, New Jersey, USA). In‐house urinalyses, including USG measurement by refractometry, dipstick chemistry and microscopic urine sediment examination were performed on the day of collection. Fibroblast growth factor‐23 (FGF23) and intact parathyroid hormone (PTH) were measured in EDTA plasma samples stored at −80°C using a validated ELISA (FGF23 ELISA Kit, Kainos Laboratories, Tokyo, Japan)^19^ and validated 2‐site immunoenzymatic assay (ST AIA‐PACK Intact PTH, Tosoh Bioscience, Tessenderlo, Belgium),^20^ respectively. A PTH concentration of 0.55 pg/mL was assigned to samples measured at <1.1 pg/mL, the lower limit of detection.^20^

### 2.4 Renal ultrasonography

Renal ultrasound examinations were carried out at baseline and visit 4 (32‐36 weeks). All ultrasound examinations were performed in lateral recumbency by the same operator (Dr P‐K Tang) using a 12 L‐RS linear transducer with a 5‐13 MHz frequency range (Logiq e R7 console, GE HealthCare, Buckinghamshire, UK). All images were saved in digital imaging and communications in medicine (DICOM) format. For each kidney, both sagittal and transverse planes were evaluated, with color Doppler used to identify the arcuate blood vessels for locating the corticomedullary junction, especially in kidneys with poor corticomedullary differentiation.

All ultrasound images and cine loop clips were blindly reviewed by the same residency‐trained radiologist (Dr D.H.N van den Broek), unaware of the cats' clinical and calcium status. Ultrasonographic variables obtained are presented in Table 1. Medullary rim sign was defined as the presence of a curvilinear hyperechoic band located in the outer renal medulla, parallel to the corticomedullary junction, that did not cast an acoustic shadow.^21^, ^22^, ^23^, ^24^ Nephrocalcinosis was defined as identification of calcification within the renal parenchyma, outside of the collecting system without overt evidence of ureteral obstruction.^25^, ^26^ Nephrolithiasis was defined as the identification of calcification within the collecting system.^1^ Renal mineralization (both nephrocalcinosis and nephrolithiasis) was detected by the presence of a hyperechoic area with acoustic shadowing.^27^ When a hyperechoic area was identified but no acoustic shadowing was evident, presence of renal mineralization was classified as “suspected.” Renal cortical scarring was defined by focal or generalized indentations of the cortical outline.^28^, ^29^

**TABLE 1:** Ultrasonographic criteria.

Criterion	Value recorded
Renal shape	Normal; irregular/rounded
Renal length (sagittal plane)	mm
Renal pelvic width (transverse plane)	mm
Cortical thickness (sagittal plane)	mm
Corticomedullary differentiation	Maintained; poorly maintained; loss
Medullary rim sign	Absent; present
Renal mineralization (nephrocalcinosis)	Absent; present (suspected); present
Renal mineralization (nephrolithiasis)	Absent; present (suspected); present
Cortical scars	Absent; present
Cortical cysts	Absent; present
Perirenal cysts	Absent; present
Perirenal fluid	Absent; present
Perirenal hyperechoic fat	Absent; present
Cystolith	Absent; single; multiple; sediment
Cystitis	Absent; present

### 2.5 Statistical analysis

Statistical analyses were performed using R software (Version 4.3.1 GUI 1.79 Big Sur ARM build, R Foundation for Statistical Computing, Vienna, Austria). Type I error rate was set at .05. The normality of continuous variables was assessed by visual inspection of Q‐Q plots and using the Shapiro‐Wilk test. Levene's test was used to test if the groups had equal variances. Most data were not normally distributed and therefore numerical data are presented as median (25th, 75th percentile). Categorical data are presented as percentages.

#### 2.5.1 Clinicopathological and ultrasonographical variables between cats with hypercalcemia and normocalcemia

Baseline variables at enrollment were compared between groups (hypercalcemia vs normocalcemia) by either independent samples *t* test or Mann‐Whitney *U* test for continuous variables with normal or skewed distributions, respectively. Chi‐squared test or Fisher's exact test was used to compare the proportions of categorical outcomes with expected counts ≥5 in all cells or <5 in any cells, respectively.

#### 2.5.2 Risk factors associated with ultrasound‐diagnosed nephrocalcinosis

Plasma concentrations of FGF23 and PTH were log‐transformed (natural logarithm [ln]) for normalization and BCS was categorized into 3 levels (1‐3, 4‐6, or 7‐9) before analysis. Enrolled cats were classified into 2 groups: “presence of nephrocalcinosis” for cats with confirmed or suspected nephrocalcinosis or “absence of nephrocalcinosis” for cats with no evidence of nephrocalcinosis on ultrasound examination.

Binary logistic regression was performed to explore baseline risk factors at enrollment associated with ultrasound‐diagnosed nephrocalcinosis in CKD cats. For univariable analysis, variables were entered as continuous variables, except for sex, BCS, MCS, consumption of a PRD and hypertension status, which were entered as categorical variables. Data available for <50% of the cats, such as blood urea nitrogen concentration (n = 15) and plasma chloride concentration (n = 15), were excluded from analyses. Variables associated with nephrocalcinosis at *P* ≤ .1 were entered into a multivariable model. The final model was derived by manual backward elimination, with *P* ≤ .05 denoting significance. The Hosmer‐Lemeshow test was used to assess the goodness‐of‐fit of the final model, and variance inflation factor was used to check for co‐linearity among the significant risk factors (*P* ≤ .05). The predictive performance of the final multivariable model was assessed by receiver operating characteristic (ROC) curve analysis. Results are reported as odds ratio (OR; 95% CI).

#### 2.5.3 Changes in clinicopathological variables in relation to presence of nephrocalcinosis

The effects of the presence of ultrasound‐diagnosed nephrocalcinosis on longitudinal biochemical data and SBP measurements from baseline and all follow‐up visits during the study period (approximately 1 year) were assessed using linear mixed effects models (R packages <lme4> and <ImerTest>). Generalized linear mixed model with cumulative logistic link function (R package <ordinal>) was used to assess the change in ordinal variables (ie, BCS and MCS) over time. Body condition score was categorized into 2 levels (1‐3, 4‐9) before analysis because of the low number of visits (n = 5; 2.8%) with BCS >6. Group (presence of nephrocalcinosis vs absence of nephrocalcinosis), time (in months [30.4 days]) and the interaction between group and time were treated as fixed effects. Cat was included as random effect to account for repeated observations from individual cats in both linear and generalized linear mixed effects models analyses. Residuals were assumed to be independent in the model, and the normality of the residuals from the linear mixed effects model was assessed by histogram visual inspection. Because of limited longitudinal data, time nested within individual cats was not included as a random effect because of a model convergence issue. No attempt was made to impute missing data. Results are reported as coefficient (*β*) ± SE.

#### 2.5.4 Comparison of ultrasonographic variables between baseline and repeated ultrasound scans

The difference in paired continuous variables (ie, renal length and cortical thickness) between baseline and repeated ultrasound scans was determined by either a paired *t* test or Wilcoxon signed ranked test based on data distribution. The difference in change in renal length (delta [Δ] renal length) and the change in cortical thickness (delta [Δ] cortical thickness) was compared between groups (normocalcemia vs hypercalcemia) using the independent samples *t* test. Results are reported as mean ± SD. For the change in paired categorical variables with dichotomous nominal outcomes (ie, renal shape and medullary rim sign) between baseline and repeated ultrasound scans, marginal homogeneity was tested using the McNemar test. Wilcoxon signed‐rank test was used to evaluate the change in paired variables with ordinal outcomes (ie, corticomedullary definition and nephrocalcinosis) between baseline and repeated ultrasound scans. Paired data are presented in contingency tables.

## 3 RESULTS

**FIGURE 1:**
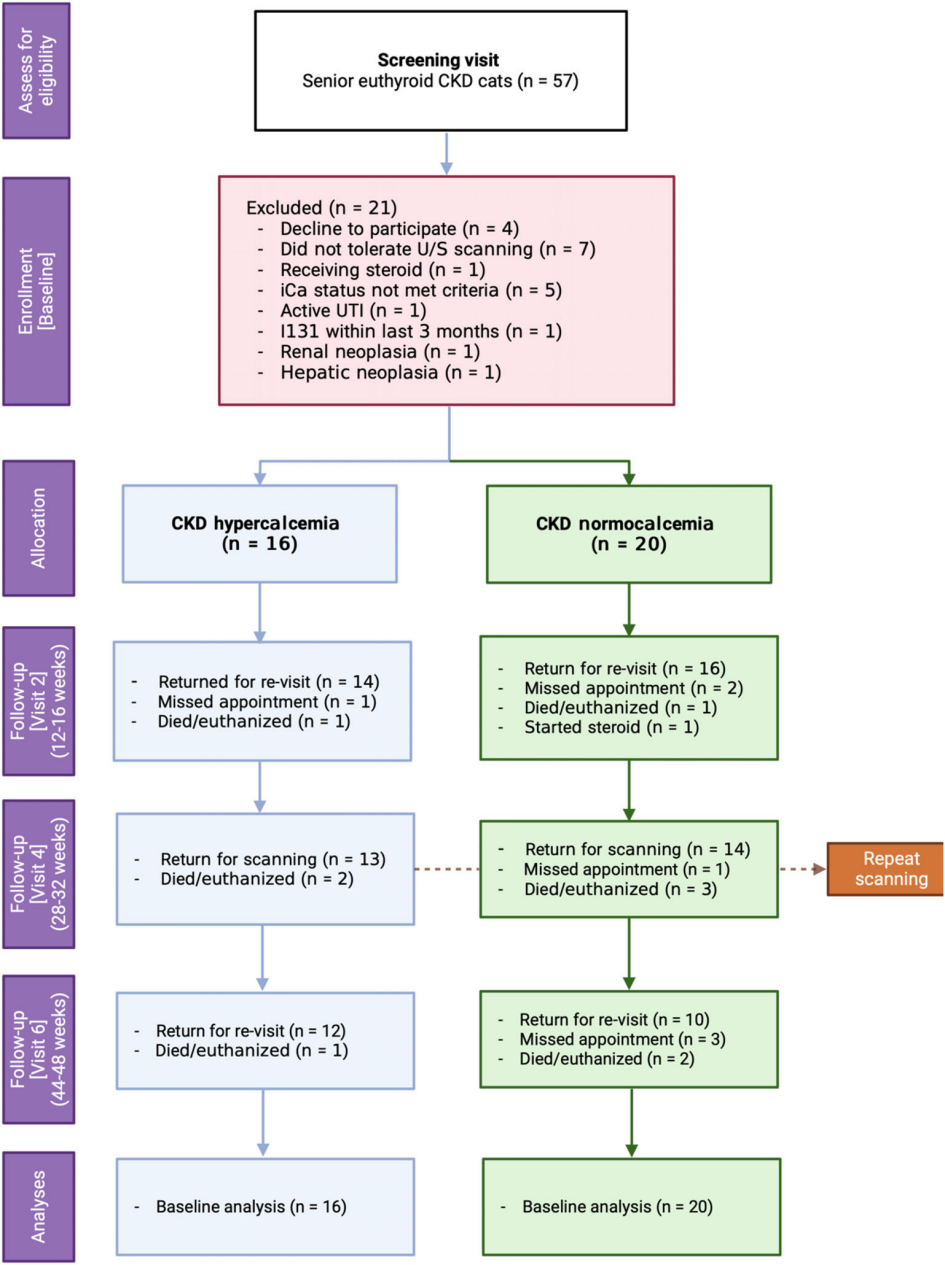
A CONSORT flow diagram of this prospective imaging study. (Created with BioRender.com)

A consolidated standards of reporting trials (CONSORT) flow diagram illustrating progress through different the phases of the study is presented in Figure 1. Fifty‐seven CKD cats were screened for eligibility, of which 21 were excluded (Figure 1) leaving 36 CKD cats that met the inclusion and exclusion criteria for enrollment. Sixteen and 20 cats were included in the hypercalcemic and normocalcemic groups, respectively.

At inclusion, 1 cat had IRIS stage 1, 25 had IRIS stage 2, 9 had IRIS Stage 3 and 1 had IRIS stage 4 CKD. Domestic shorthair was the most common breed (n = 24), followed by domestic longhair (n = 6), Birman (n = 3), and 1 each of the following: Bengal, Burmese and Devon Rex. Sixty‐seven percent (n = 24) of CKD cats were eating a PRD at enrollment, 19% (n = 7) were eating a MPRD, and 14% (n = 5) were consuming maintenance diets because a diagnosis of CKD was confirmed on the day of enrollment (n = 4) or the cat did not accept a PRD (n = 1). Of the 24 CKD cats eating a PRD (13 with and 11 without nephrocalcinosis), the majority were eating exclusively PRD (100% [75%, 100%]) at enrollment. Of the 7 cats eating a MPRD, 6/7 had nephrocalcinosis. Of the 4 newly diagnosed CKD cats eating maintenance diets, 3/4 had nephrocalcinosis. No difference in diet fed at enrollment was found between groups (*P* = .34). During the study period, 2 cats (1 in each nephrocalcinosis group) had diet changed from PRD to MPRD because of development of hypercalcemia, and all 4 newly diagnosed CKD cats had diet changed to a PRD and continued throughout. Furthermore, 6 cats (4 with and 2 without nephrocalcinosis) received PO magnesium supplementation for a median of 77 (range, 42‐116) days as part of a different prospective study.

Of the 36 CKD cats enrolled, 22 cats (61%) had ultrasonographically‐diagnosed nephrocalcinosis, whereas 14 cats (39%) had no evidence of nephrocalcinosis. The time interval between CKD diagnosis and renal ultrasonography at enrollment was a median of 101 (range, 0‐1777) days, with no difference between cats with and without nephrocalcinosis (*P* = .3). Ten cats (4 hypercalcemic and 6 normocalcemic) died or were euthanized during the study, a median of 178 (range, 27‐288) after enrollment. Of these 10 cats, 8 cats had evidence of ultrasound‐diagnosed nephrocalcinosis (4 with bilateral and 4 with unilateral nephrocalcinosis) at baseline. The study had a median follow‐up period of 322 (range, 0‐511) days.

### 3.1 Comparison of cats with differing calcium status

Baseline clinicopathological variables are summarized in Table 2. A higher proportion of CKD cats with hypercalcemia had evidence of nephrocalcinosis in 1 or both kidneys compared with those with normocalcemia (81% vs 45%; *P* = .04). Hypercalcemic CKD cats had significantly higher plasma albumin concentrations (3.2 [3.1, 3.3] vs 2.9 [2.8, 3.1] g/dL; *P* = .01) and lower plasma PTH concentrations (5.1 [.6, 13.3] vs 20.9 [8.9, 55.4] pg/mL; *P* = .003) than normocalcemic CKD cats. No other significant differences in clinicopathological (Table 2) or ultrasonographic variables (Supplementary Table 1) were identified based on calcium status.

**TABLE 2:** Descriptive statistics on clinicopathological variables at enrolment for CKD cats in this prospective imaging study, grouped according to the ionized calcium status (“CKD normocalcemia” vs “CKD hypercalcemia”).

Variables (reference interval)	CKD normocalcemia (n = 20)		CKD hypercalcemia (n = 16)		*P*‐value
	Median [25th, 75th percentile]	n	Median [25th, 75th percentile]	n	
Total calcium (8.2‐11.8 mg/dL)	9.86 [9.75, 10.09]	20	10.9 [10.5, 11.66]	16	**<.001**
Ionized calcium (4.76‐5.48 mg/dL)	5.28 [5.23, 5.37]	20	5.7 [5.67, 6.19]	16	**<.001**
Age (years)	14.6 [13.2, 16.6]	20	14.5 [12.6, 16.7]	16	.73
BCS (“1‐3,” “4‐6,” “7‐9,” n [%])	9 [45], 11 [55], 0 [0]	20	3 [19], 12 [75], 1 [6]	16	.16
MCS (“0,” “1,” “2,” “3,” n [%])	1 [5], 6 [30], 13 [66], 0 [0]	20	2 [13], 1 [7], 11 [73], 1[7]	15	.21
Weight (kg)	3.4 [3.1, 4.2]	20	3.8 [3.2, 4.6]	16	.24
Sex (female neutered, n [%])	9 [45]	20	9 [56]	16	.5
Albumin (2.5‐4.5 g/dL)	2.9 [2.8, 3.1]	20	3.2 [3.1, 3.3]	16	**.01**
ALP (≤ 60 U/L)	29 [20, 34]	20	23 [20, 25]	16	.19
ALT (5‐60 U/L)	50 [38, 60]	20	49 [37, 59]	16	.82
CaPP (<70 mg^2^/dL^2^)	38.3 [33.9, 45.5]	20	42.8 [37.4, 51.7]	16	.09
Chloride (100‐124 mEq/L)	119 [115, 120]	9	119 [117126]	6	.20
Creatinine (0.23‐2 mg/dL)	2.53 [2.18, 3.07]	20	2.24 [2.00, 2.83]	16	.29
FGF23^a^ (56‐700 pg/mL)	391 [265, 852]	18	756 [326, 1983]	15	.22
Glucose (54‐117 mg/dL)	106 [93, 130]	20	114 [106, 129]	16	.31
Venous HCO_3_^−^ (17‐24 mEq/L)	22.5 [19.7, 23.6]	20	21.0 [8.5, 22.0]	16	.1
Hypertension (controlled) (n [%])	2 [10]	20	2 [12.5]	16	1
PCV (30‐45%)	34 [28, 37]	20	32 [30, 34]	16	.57
Venous pH (7.21‐7.44)	7.38 [7.35, 7.4]	20	7.37 [7.29, 7.37]	16	.07
Phosphate (2.79‐6.81 mg/dL)	3.90 [3.42, 4.38]	20	3.75 [3.53, 4.35]	16	.96
Potassium (3.5‐5.5 mEq/L)	3.9 [3.4, 4.1]	20	3.7 [3.5, 4.0]	16	.63
PTH^a^ (2.6‐17.6 pg/mL)	20.9 [8.9, 55.4]	17	5.1 [0.6, 13.3]	14	**.003**
SBP (<160 mm Hg)	128 [120, 143]	20	124 [121, 133]	16	.38
SDMA (1‐14 μg/dL)	19 [17, 25]	20	17 [14, 24]	16	.41
Sodium (145‐157 mEq/L)	152 [150, 152]	20	151 [149, 151]	16	.52
Total magnesium (1.73‐2.57 mg/dL)	2.05 [1.91, 2.19]	20	2.01 [1.79, 2.24]	16	.44
Total protein (6.0‐8.0 g/dL)	7.9 [7.3, 8.5]	20	7.5 [7.2, 8.0]	16	.52
Urea (7.0‐27.7 mg/dL)	47.3 [42.9 51.5]	9	44.3 [36.6, 50.7]	6	.6
USG (≥1.035)	1.016 [1.015, 1.017]	14	1.017 [1.015, 1.022]	9	.42
Urine pH	6.0 [5.0, 6.8]	14	6.0 [5.0, 6.0]	9	.66
Nephrocalcinosis (“Y,” n [%])	9 [45]	20	13 [81]	16	**.04**
Of which suspected	3 [30]	9	7 [54]	13	.42

*Note*: Significant difference between groups (*P* ≤ .05) are highlighted in bold.

Abbreviations: ALP, alkaline phosphatase; ALT, alanine aminotransferase; BCS, body condition score; CaPP, calcium phosphate product; FGF23, fibroblast growth factor‐23; HCO_3_^−^, bicarbonate; MCS, muscle condition score; n, number of cats; PCV, packed cell volume; PTH, parathyroid hormone; SBP, systolic blood pressure; SDMA, symmetric dimethylarginine; USG, urine specific gravity.

^a^ Baseline FGF23 and PTH were log‐transformed for comparison using independent samples *t*‐test and Mann‐Whitney *U* test, respectively.

### 3.2 Risk factors associated with ultrasound‐diagnosed nephrocalcinosis

Baseline clinicopathological and ultrasonographic variables between CKD cats with and without nephrocalcinosis are summarized in Tables 3 and 4, respectively. The CKD cats with nephrocalcinosis had higher plasma concentrations of tCa, calcium phosphate product (CaPP), albumin and total magnesium concentrations and alanine aminotransferase (ALT) activity compared with CKD cats without nephrocalcinosis. A significantly higher proportion of CKD cats with nephrocalcinosis had unilateral loss of corticomedullary differentiation compared to those without (50% vs 14%; *P* = .02).

**TABLE 3:** Descriptive statistics on clinicopathological variables at enrolment for CKD cats in this prospective imaging study, grouped according to the detection of nephrocalcinosis by ultrasonography (“Absence of nephrocalcinosis” vs “Presence of nephrocalcinosis”).

Variables (reference interval)	Absence of nephrocalcinosis (n = 14)		Presence of nephrocalcinosis (n = 22)		*P*‐value
	Median [25th, 75th percentile]	n	Median [25th, 75th percentile]	n	
Total calcium (8.2‐11.8 mg/dL)	9.86 [9.77, 10.06]	14	10.74 [10.08, 11.16]	22	**.01**
Ionized calcium (4.76‐5.48 mg/dL)	5.30 [5.25, 5.44]	14	5.62 [5.30, 5.81]	22	.08
Age (years)	15.1 [13.5, 16.7]	14	14.1 [12.5, 16.4]	22	.22
BCS (“1‐3,” “4‐6,” “7‐9,” n [%])	4 [29], 10 [71], 0 [0]	14	8 [36], 13 [59], 1 [5]	22	.83
MCS (“0,” “1,” “2,” “3,” n [%])	1 [7], 3 [21], 10 [71], 0 [0]	14	2 [10], 4 [19], 14 [67], 1 [5]	21	1
Weight (kg)	3.2 [3.1, 3.7]	14	3.8 [3.3, 4.4]	22	.13
Sex (female neutered, n [%])	8 [57]	14	10 [45]	22	.49
Albumin (2.5‐4.5 g/dL)	2.9 [2.8, 3.1]	14	3.1 [3.0, 3.3]	22	**.03**
ALP (≤ 60 U/L)	26 [20, 34]	14	24 [20, 30]	22	.66
ALT (5‐60 U/L)	43 [32, 49]	14	52 [42, 64]	22	**.03**
CaPP (<70 mg^2^/dL^2^)	34.9 [32.8, 39.8]	14	42.8 [38.1, 53.2]	22	**.01**
Chloride (100‐124 mEq/L)	117 [115, 118]	7	121 [118125]	8	.07
Creatinine (0.23‐2 mg/dL)	2.27 [2.05, 2.48]	14	2.60 [2.08, 3.09]	22	.14
FGF23^a^ (56‐700 pg/mL)	383 [265, 657]	12	776 [289, 1988]	21	.06
Glucose (54‐117 mg/dL)	117 [97, 141]	14	110 [93, 124]	22	.16
Venous HCO_3_^−^ (17‐24 mmol/L)	21.9 [19.8, 23.0]	14	21.0 [18.8, 22.6]	22	.4
Hypertension (controlled) (n [%])	2 [14]	14	2 [9]	22	.63
PCV (30‐45%)	34 [32, 37]	14	31 [28, 35]	22	.13
Venous pH (7.21‐7.44)	7.38 [7.35, 7.4]	14	7.37 [7.30, 7.38]	22	.24
Phosphate (2.79‐6.81 mg/dL)	3.55 [3.20, 3.95]	14	4.06 [3.72, 4.68]	22	.06
Potassium (3.5‐5.5 mmol/L)	3.5 [3.4, 4]	14	3.9 [3.6, 4.2]	22	.15
PTH^a^ (2.6‐17.6 pg/mL)	8.9 [5.0, 18.5]	11	15.8 [4.3, 55.7]	20	.54
SBP (<160 mm Hg)	126 [120, 149]	14	124 [120, 138]	22	.35
SDMA (1‐14 μg/dL)	18 [14, 20]	14	20 [15, 25]	22	.3
Sodium (145‐157 mEq/L)	152 [149, 152]	14	151 [149, 152]	22	.88
Total magnesium (1.73‐2.57 mg/dL)	1.93 [1.85, 2.08]	14	2.15 [1.88, 2.40]	22	**.02**
Total protein (6.0‐8.0 g/dL)	7.9 [7.4, 8.5]	14	7.4 [7.2, 8.2]	22	.23
Urea (7.0‐27.7 mg/dL)	41.2 [31.9, 45.1]	7	49.4 [46.7, 53.6]	8	.06
USG (≥1.035)	1.016 [1.015, 1.018]	11	1.016 [1.016, 1.019]	12	.38
Urine pH	6 [5, 7]	11	6 [5, 6]	12	.49
CKD duration (days)	71 [38, 152]	14	106 [71, 688]	22	.17

*Note*: Significant difference between groups (*P* ≤ .05) are highlighted in bold.

Abbreviations: ALP, alkaline phosphatase; ALT, alanine aminotransferase; BCS, body condition score; CaPP, calcium phosphate product; FGF23, fibroblast growth factor‐23; HCO_3_^−^, bicarbonate; MCS, muscle condition score; n, number of cats; PCV, packed cell volume; PTH, parathyroid hormone; SBP, systolic blood pressure; SDMA, symmetric dimethylarginine; USG, urine specific gravity.

^a^ Baseline FGF23 and PTH were log‐transformed for comparison using independent samples *t* test.

**TABLE 4:** Descriptive statistics on baseline ultrasonographical variables for CKD cats enrolled in this prospective imaging study, grouped according to the detection of macro‐nephrocalcinosis by ultrasonography (“Absence of nephrocalcinosis” vs “Presence of nephrocalcinosis”).

Variables	Absence of nephrocalcinosis (n = 14)		Presence of nephrocalcinosis (n = 22)		*P*‐value
	Median [25th, 75th percentile]	n	Median [25th, 75th percentile]	n	
Renal shape (left kidney)	Normal, 9; Irregular, 5	14	Normal, 14; Irregular, 8	22	.97
Renal shape (right kidney)	Normal, 10; Irregular, 4	14	Normal, 13; Irregular, 9	22	.5
Renal length (left kidney, cm)	3.1 [2.6, 3.4]	14	3.1 [2.9, 3.4]	21	.27
Renal length (right kidney, cm)	3.2 [2.9, 3.5]	14	3.1 [2.6, 3.5]	21	.44
Corticomedullary differentiation (left kidney)	Maintained, 2; Poorly maintained, 9; Loss, 3	14	Maintained, 1; Poorly maintained, 8; Loss, 13	22	.08
Corticomedullary differentiation (right kidney)	Maintained, 0; Poorly maintained, 12; Loss, 2	14	Maintained, 2; Poorly maintained, 9; Loss, 11	22	**.02**
Cortical thickness (left kidney, cm)	0.34 [0.30, 0.45]	14	0.40 [0.38, 0.47]	21	.27
Cortical thickness (right kidney, cm)	0.37 [0.35, 0.45]	14	0.33 [0.29, 0.42]	18	.17
Medullary rim sign (left kidney)	Present, 2; Absent, 12	14	Present, 4; Absent, 17	21	1
Medullary rim sign (right kidney)	Present, 2; Absent, 12	14	Present, 3; Absent, 18	21	1
Nephrolithiasis (left kidney)	Present, 0; Absent, 14	14	Present, 4; Absent, 17	21	.13
Nephrolithiasis (right kidney)	Present, 0; Absent, 14	14	Present, 1; Absent, 20	21	1
Other parenchymal abnormality (left kidney)	Cortical scars, 2; Cortical cysts, 2; None, 10	14	Cortical scars, 1; Cortical cysts, 8; None, 12	21	.24
Other parenchymal abnormality (right kidney)	Cortical scars, 2; Cortical cysts, 0; None, 12	14	Cortical scars, 1; Cortical cysts, 4; None, 16	21	.2
Perirenal abnormality (left kidney)	None, 14	14	None, 21	21	1
Perirenal abnormality (right kidney)	None, 14	14	None, 21	21	1

*Note*: Significant difference between groups (*P* ≤ .05) are highlighted in bold.

Abbreviation: n, number of cats.

Results from the univariable and multivariable logistic regression models are shown in Table 5. In the final multivariable model (n = 36), plasma concentrations of iCa (OR, 1.27; 95% CI, 1.05‐1.73) per 0.1 mg/dL increase (*P* = .01), phosphate (OR, 1.16; 95% CI, 1.00‐1.43) per 0.1 mg/dL increase (*P* = .05), and creatinine (OR, 1.29; 95% CI, 1.03‐1.85) per 0.1 mg/dL increase (*P* = .02) and ALT activity (OR, 2.08; 95% CI, 1.04‐5.61) per 10 U/L increase (*P* = .04) remained independent risk factors for ultrasound‐diagnosed nephrocalcinosis. The area under the ROC curve was 0.88 (95% CI, 0.77‐0.99), suggesting the final multivariable model had excellent predictive accuracy.

**TABLE 5:** Univariable and multivariable logistic regression to identify risk factors at enrolment associated with ultrasound‐diagnosed nephrocalcinosis in CKD cats (n = 36).

Variables	Univariable analysis		Multivariable analysis	
	OR (95% CI)	*P*‐value	OR (95% CI)	*P*‐value
Total calcium (mg/dL * 10)	1.12 (1.02‐1.28)	**.01**		
Ionized calcium (mg/dL * 10)	1.15 (0.99‐1.47)	.06	1.27 (1.05‐1.73)	**.01**
Phosphate (mg/dL * 10)	1.10 (1.00‐1.26)	**.04**	1.16 (1.00‐1.43)	**.05**
Creatinine (mg/dL * 10)	1.12 (1.00‐1.30)	**.04**	1.29 (1.03‐1.85)	**.02**
Albumin (g/dL * 10)	1.46 (1.09‐2.15)	**.01**		
Total magnesium (mg/dL * 10)	1.30 (1.02‐1.78)	**.04**		
ALT (U/L * 0.1)	1.79 (1.12‐3.38)	**.01**	2.08 (1.04‐5.61)	**.04**

*Note*: Significant variables (*P* ≤ .05) in univariable and multivariable models are highlighted in bold.

Abbreviations: ALT, alanine transaminase; CI, confidence interval; OR, odds ratio.

### 3.3 Changes in clinicopathological variables over time in relation to ultrasound‐diagnosed nephrocalcinosis

**FIGURE 2:**
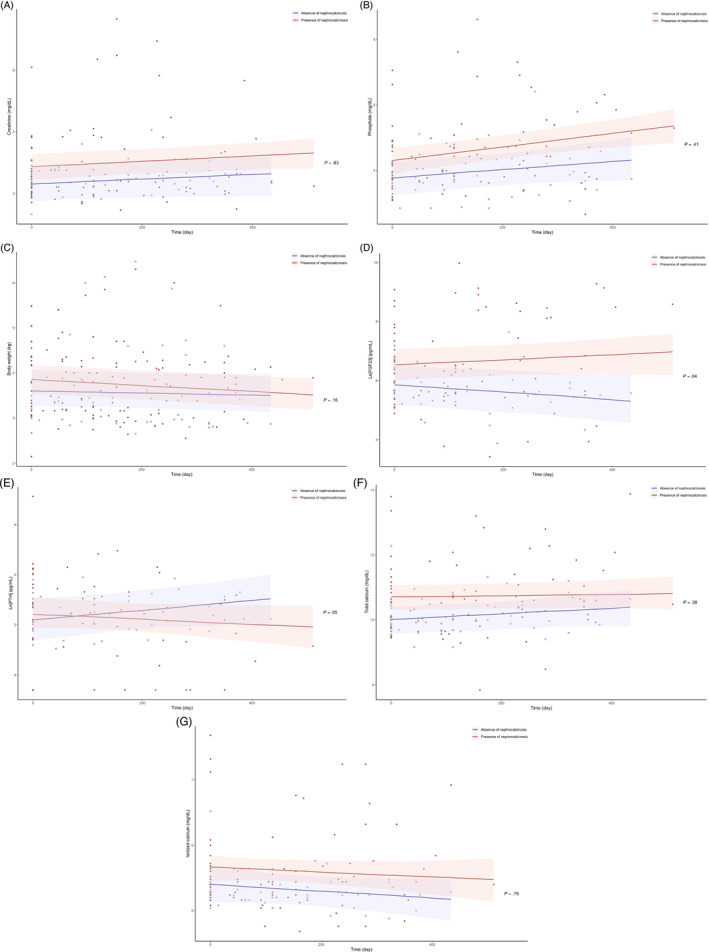
Scatter plots illustrating the linear change of plasma (A) creatinine; (B) phosphate; (C) body weight; (D) ln[FGF23]; (E) ln[PTH]; (F) total calcium; and (G) ionized calcium in all enrolled cats in this prospective imaging study (n = 36) according to the classification of nephrocalcinosis determined by ultrasonography at enrolment (“absence of nephrocalcinosis” [crosses] vs “presence of nephrocalcinosis” [dots]) during the study period. The *P*‐value refers to the Group*Time interaction (as shown in Table 6) analyzed using linear mixed effects models, which assessed the difference in rate of change of the outcome variable between groups (“absence of nephrocalcinosis” vs “presence of nephrocalcinosis”) over time.

No significant difference was found in the rate of change in plasma concentrations of creatinine and phosphate between CKD cats with and without nephrocalcinosis (Table 6 and Supplementary Table 2), but plasma concentrations of creatinine and phosphate increased significantly over time in cats with nephrocalcinosis (creatinine, *β*, .03 ± .01 mg/dL per month, *P* = .04; phosphate, *β*, .06 ± .02 mg/dL per month, *P* < .001), whereas these variables remained relatively stable over time in those without nephrocalcinosis (creatinine, *β*, .02 ± .02 mg/dL per month, *P* = .2; phosphate, *β*, .04 ± .02 mg/dL per month, *P* = .07; Figure 2A,B). No significant difference in rate of change in body weight was observed between CKD cats with and without nephrocalcinosis (Table 6). However, body weight was found to significantly decrease over time in cats with nephrocalcinosis (*β*, .02 ± .01 kg per month, *P* < .001) but not in those without nephrocalcinosis (*β*, .01 ± .01 kg per month; *P* = .32; Figure 2C).

**TABLE 6:** Linear mixed model and generalized linear mixed model analyses examining the change in clinicopathological variables over time in all enrolled CKD cats (n = 36) during the study period.

Variables	Group	Time	Group*Time
BCS (“1‐3,” “4‐9”)	.77	.06	.39
MCS (“0,” “1,” “2,” “3”)	.79	.06	.41
Body weight (kg)	.37	**.003**	.16
Albumin (g/dL)	**.04**	.25	.06
ALP (U/L)	.93	.21	.23
ALT (U/L)	**.02**	.32	.52
Creatinine (0.23‐2 mg/dL)	.14	**.03**	.83
ln[FGF23] (pg/mL)	.12	.72	**.04**
Venous HCO_3_^−^ (mEq/L)	.5	.81	.6
Ionized calcium (mg/dL)	.11	.13	.79
PCV (%)	.19	.14	.13
Venous pH	.43	.22	.54
Phosphate (mg/dL)	.12	**<.001**	.41
Potassium (mEq/L)	.11	.98	.11
ln[PTH] (pg/mL)	.62	.61	**.05**
SBP (mm Hg)	.41	.4	.46
SDMA (μg/dL)	.22	.22	.72
Sodium (mEq/L)	.07	.33	.16
Total calcium (mg/dL)	**.03**	.15	.38
Total magnesium (mg/dL)	.21	.17	.46
Total protein (g/dL)	.1	.69	.95

*Note*: Summary of *P*‐values for all variables included in the model. Group represents cats in “absence of nephrocalcinosis” or “presence of nephrocalcinosis” group based on the ultrasonographic findings at enrolment. Outcome variables showing significant change over time and between groups (*P* ≤ .05) are highlighted in bold. The unit used for time was month (30.4 days). A significant effect in the group column indicates a significant difference between the two groups at baseline for a given parameter (the start of the regression line at time 0). A significant effect of Group*Time interaction indicates that the rate of change of the outcome variable differs significantly between groups (“absence of nephrocalcinosis” vs “presence of nephrocalcinosis”) over time. If Group*Time was not significant, and the effect of Time was significant, this indicates no difference in the rate of change of the outcome variable between groups, however, the overall gradient of the outcome variable plotted against time (with the data from all groups combined) differs significantly from zero.

Abbreviations: ALP, alkaline phosphatase; ALT, alanine aminotransferase; BCS, body condition score; HCO_3_^−^, bicarbonate; ln[FGF23], log‐transformed fibroblast growth factor‐23; ln[PTH], log‐transformed parathyroid hormone; MCS, muscle condition score; PCV, packed cell volume; SBP, systolic blood pressure; SDMA, symmetric dimethylarginine.

Although the gradients of regression lines for ln[FGF23] and ln[PTH] did not differ from zero in either group (ie, no significant change over time), the rate of change in both ln[FGF23] and ln[PTH] were found to be significantly different between the 2 groups (Table 6, Figure 2D,E). No difference was found in the rate of change in plasma total and ionized calcium concentrations between and within groups (Table 6, Figure 2F,G, and Supplementary Table 2).

### 3.4 Comparison of ultrasonographic variables between baseline and repeated ultrasound scans

Twenty‐seven cats returned for a repeated ultrasound scan, a median of 238 (range, 175‐371) days post enrollment, of which 13 cats were normocalcemic and 14 were hypercalcemic. When evaluating all 27 cats, no differences were found in renal length, cortical thickness (Supplementary Figure 2), proportion of CKD cats with irregular renal shape (Supplementary Table 3), presence of medullary rim sign (Supplementary Table 4), classification of corticomedullary definition (Supplementary Table 5), or nephrocalcinosis (Supplementary Table 6) between baseline and repeated ultrasound scans. No difference was found in the change of renal length and change in cortical thickness between hypercalcemic and normocalcemic CKD cats (data not shown).

## 4 DISCUSSION

Nephrocalcinosis, detected by ultrasonography, was identified frequently in cats with CKD, especially those with ionized hypercalcemia. A significantly higher proportion of hypercalcemic CKD cats had nephrocalcinosis (81%) compared with those that had normal calcium status (45%). Higher iCa, phosphate, creatinine and ALT results were independent risk factors for nephrocalcinosis, which appears to be associated with CKD progression as determined by increasing plasma creatinine and phosphate concentrations over time.

At enrollment, concentrations of iCa, phosphate, creatinine and ALT activity were independent risk factors for ultrasound‐diagnosed nephrocalcinosis. In a previous retrospective study, plasma tCa concentration was reported as an independent risk factor for microscopic nephrocalcinosis at necropsy in CKD cats,^5^ but measurement of iCa had not been performed. Plasma tCa was also a significant risk factor for nephrocalcinosis in univariable analysis in our study, but was removed from the multivariable analysis when iCa was entered. This seems logical, given that blood iCa is the physiologically active form of calcium.^30^, ^31^ Ionized hypercalcemia also has been associated with nephrocalcinosis in human patients.^32^, ^33^, ^34^

Increased plasma phosphate concentration was another independent risk factor for nephrocalcinosis, which is consistent physiologically because calcium binds with phosphate to form hydroxyapatite and this process is a fundamental factor in soft tissue mineralization. An association between hyperphosphatemia and nephrocalcinosis was previously reported in FGF23‐knockout mice.^35^ Complete resolution of nephrocalcinosis was achieved after dietary phosphate restriction and lowering of serum phosphate concentration.^35^

Nephrocalcinosis was detected in 61% of our cats with CKD and a similar prevalence (50‐78%) in cats with CKD was documented previously in 2 retrospective studies.^5^, ^13^ In our study, CKD cats with more advanced azotemia also appeared to be at greater risk for nephrocalcinosis. This pathological feature is common in human patients with CKD, with increasing prevalence as kidney function decreases. Fifty‐four percent of human patients with stage 5 CKD had microscopic nephrocalcinosis, compared with 14.3% and 4.6% reported in stage 1‐2 CKD patients and non‐azotemic kidney donors, respectively.^4^, ^14^, ^16^ In accordance with our results, a previous study involving 211 human patients with CKD of different stages identified an independent association between serum creatinine concentration and nephrocalcinosis, with higher estimated glomerular filtration rate measured in those without nephrocalcinosis.^4^ Furthermore, a study in humans found a strong correlation between serum creatinine concentration and the degree of vascular calcification, and uremic serum was found to induce calcification in cultured smooth muscle cells.^36^

Increased plasma ALT activity also was associated with increased risk of nephrocalcinosis in cats with CKD. Alanine aminotransferase is a marker of hepatocellular damage.^37^ This finding is interesting but seems unlikely to be clinically relevant. Seventy‐eight percent (28/36) of CKD cats in our study had plasma ALT activity within the reference range (≤60 U/L). When plasma ALT activity was transformed into a categorical variable based on the respective reference interval (≤60 U/L vs >60 U/L), the univariable binomial logistic regression analysis indicated that ALT activity was not significantly associated with risk of nephrocalcinosis (*P* = .35). However, a possible explanation for this result could be related to the accumulation of calciprotein particles (CPP). Primary CPP (CPP‐1) are predominantly cleared by liver sinusoidal endothelial cells^38^ and CPP‐1 spontaneously transform into secondary CPP (CPP‐2) over time which are capable of inducing inflammation and calcification.^39^, ^40^ Therefore, nephrocalcinosis may be a consequence of prolonged CPP presence and clearance.^41^ Additional studies are required to better understand the interplay between ALT activity and nephrocalcinosis in relation to CPP.

In our study, a higher proportion of CKD cats with ultrasound‐diagnosed nephrocalcinosis had loss of corticomedullary differentiation in at least 1 kidney at enrollment. Increased renal cortical echogenicity and loss of corticomedullary differentiation are common ultrasonographic findings in human patients with chronic ESRD, with prevalence of 100% and 85%, respectively, documented in 1 study.^42^ In an another retrospective study involving human patients undergoing ultrasound‐guided renal biopsy, renal cortical echogenicity was positively correlated with chronic renal pathology including interstitial inflammation, interstitial fibrosis, tubular atrophy and glomerular sclerosis.^43^ A prospective study also identified a significant association between renal cortical echogenicity and severity of azotemia in human CKD patients.^44^ Consistent with these findings in humans, both ex vivo^45^ and in vivo^46^ studies in cats identified a positive relationship between renal cortical echogenicity and severity of degenerative changes in kidney samples obtained at necropsy. Furthermore, 10 (45%) CKD cats with nephrocalcinosis, compared with only 2 (14%) without nephrocalcinosis, had complete loss of corticomedullary differentiation detected in both kidneys, suggesting that nephrocalcinosis is associated with loss of corticomedullary differentiation and CKD progression in cats. This conclusion is further supported by the independent association found between plasma creatinine concentration and presence of nephrocalcinosis in our study.

Significant increases in plasma creatinine and phosphate concentrations over time were observed in CKD cats with nephrocalcinosis at enrollment. In contrast, these variables remained comparatively stable over time in cats without nephrocalcinosis, suggesting a deleterious effect of nephrocalcinosis on accelerating renal functional deterioration in CKD cats. Nevertheless, the differences in rate of change in plasma creatinine and phosphate concentrations between the 2 nephrocalcinosis groups were not significant, which could be attributed to the relatively small number of cats included in our study for complex longitudinal comparison. In humans, nephrocalcinosis previously has been associated with increased risk of renal failure,^9^ and a positive relationship exists between renal calcium content and serum creatinine concentration.^47^ Increased kidney deposition of calcium is associated with more severe interstitial inflammation and renal dysfunction in uremic rat models.^48^ Similar findings were documented in a study of cats with CKD.^13^ Furthermore, in our study, 8 CKD cats (36%) with nephrocalcinosis died or were euthanized during the first 365 days post enrollment (7 because of renal diseases and 1 because of non‐renal diseases), compared with 2 CKD cats (14%) without nephrocalcinosis (both unknown cause). These results further support our hypothesis that nephrocalcinosis is associated with CKD progression, and potentially the risk of death in cats. Interestingly, this finding is contrary to a previous retrospective study that suggested that microscopic nephrocalcinosis was not associated with accelerated decrease in renal function.^5^ This discrepancy could be attributed to the different timepoints (ante‐mortem vs post‐mortem) and methods applied (ultrasonography vs histopathology) for the detection of nephrocalcinosis, and differences in study designs (prospective vs retrospective). Survival analyses, such as Cox proportional hazards model and Kaplan‐Meier curve, are required to further evaluate the association between nephrocalcinosis and all‐cause mortality when longer follow‐up data from the enrolled cats become available. Interestingly, nephrolithiasis was identified in 11% (n = 4) of our cats, with 3 in the hypercalcemic group and 1 in the normocalcemic group. Calculus analysis was only available for 1 of these cats and confirmed the nephrolith to be 100% calcium oxalate monohydrate, the most common composition for upper urinary tract uroliths in cats.^49^, ^50^, ^51^ However, none of the nephroliths in the 4 cats was associated with ureteral obstruction and therefore the nephroliths are unlikely to have contributed to the associations between nephrocalcinosis and CKD progression found in our study.

Our prospective study had some limitations. Other imaging modalities such as radiography or computed tomography (CT) were not available for the study protocol. Nonetheless, ultrasonography has been found to be superior to CT for the detection of nephrocalcinosis in humans.^1^ However, the sensitivity and specificity of such detection may be operator‐dependent. Therefore, nephrocalcinosis could have been missed and the prevalence of nephrocalcinosis could have been underestimated because of a type II error. Conversely, CKD cats with suspected nephrocalcinosis were grouped together with those with strong evidence of nephrocalcinosis for statistical analyses, and it is also possible that type I error could have occurred by misinterpreting renal fibrosis, a common pathological feature in CKD, as suspected nephrocalcinosis. Because of classification of cases in our study exploring the risk factors and effects of nephrocalcinosis on CKD‐mineral and bone disorder (CKD‐MBD) in cats was based on ultrasonographic findings at enrollment, misclassification bias could have been introduced. However, we attempt to mitigate against this possibility by having all images and cine loop clips blindly reviewed by the same residency‐trained radiologist. Therefore, the possibility of such bias was deemed to be relatively low. In addition, recent work has determined that ultrasound‐diagnosed nephrocalcinosis correlates well with microscopic nephrocalcinosis detected on histopathology.^14^ Contrary to a recent retrospective study,^5^ consumption of a PRD was not found to be a significant risk factor for nephrocalcinosis in our study, which is most likely attributed to the heterogeneity of populations between studies, with the majority of cats enrolled in our study having been eating a high proportion of PRD. Another limitation of our study is that diet was not controlled but was tailored to the individual cat, and diet could have influenced change in variables over time. Nevertheless, no significant difference in diet fed at enrollment or diet changes over time were found between the 2 nephrocalcinosis groups.

Despite the prospective nature of our study, direct causal relationships between the risk factors identified and nephrocalcinosis could not be inferred because the exact time point when nephrocalcinosis developed in these cats remains unknown. The median time interval between CKD diagnosis and baseline renal ultrasonography was 105 days, with no difference observed between cats with evidence of nephrocalcinosis and those without nephrocalcinosis. In addition, although the number of cats enrolled met the sample size required for the assessment of nephrocalcinosis prevalence between groups based on the calcium status, this may not be sufficient for the comparison of the rate of change in clinicopathological variables longitudinally between cats with and without nephrocalcinosis. As a result, the linear mixed model analyses may be subject to a type II error, especially the “absence of nephrocalcinosis” group with 14 cats.

In conclusion, our results showed that nephrocalcinosis is highly prevalent in cats with CKD, with increasing prevalence in those with ionized hypercalcemia. Increased iCa, phosphate and creatinine concentrations and ALT activity were independent risk factors for nephrocalcinosis in CKD cats. Results from our longitudinal data suggest that nephrocalcinosis contributes, at least in part, to the progression of CKD, as demonstrated by the significant increase in plasma concentrations of creatinine, phosphate and FGF23 over time in CKD cats with ultrasound‐diagnosed nephrocalcinosis at enrollment. Therefore, renal ultrasonography is recommended in CKD cats, particularly those with hypercalcemia or hyperphosphatemia, for the detection of nephrocalcinosis because doing so may provide additional prognostic information. Ultrasonography should be combined with regular measurement of iCa, and controlling calcium disturbance should be considered fundamental to the management of cats with CKD‐MBD. Further study is required to investigate the effects of calcium‐lowering treatment on nephrocalcinosis and its long‐term benefit on CKD progression and survival in cats.

## CONFLICT OF INTEREST DECLARATION

Pak Kan Tang received a PhD studentship funded by Royal Canin SAS. Rebecca Geddes received funding from Petplan, Royal Canin, an RVC Internal Grant, The Academy of Medical Sciences and The Everycat Foundation; has previously had a consultancy agreement with Boehringer Ingelheim; has received speaking honoraria from Boehringer Ingelheim, Idexx and Royal Canin. Rosanne Jepson received funding from PetPlan, Feline Foundation for Renal Research, RVC Internal Grant, PetSavers, and consultancy agreements: Boehringer Ingelheim, Merial, CEVA. Speaking honoraria: Boehringer Ingelheim, Hills Pet Nutrition, CEVA. Jonathan Elliott has Consultancy agreements with: Elanco Ltd, CEVA Animal Health Ltd, Boehringer Ingelheim Ltd, MSD Animal Health Ltd., Orion Incorp, Idexx Ltd, Waltham Petcare Science Institute, Invetx Inc and Zoetis Ltd. received grant funding from Elanco Ltd, Waltham Centre for Pet Nutrition, Royal Canin SAS, Idexx Ltd., CEVA Animal Health. He is a member of the International Renal Interest Society which receives sponsorship from Zoetis.

## OFF‐LABEL ANTIMICROBIAL DECLARATION

Authors declare no off‐label use of antimicrobials.

## INSTITUTIONAL ANIMAL CARE AND USE COMMITTEE (IACUC) OR OTHER APPROVAL DECLARATION

This study was part of a larger observational cohort for which approval of the Ethics and Welfare Committee of the Royal Veterinary College (URN 2013 1258E) had been granted.

## HUMAN ETHICS APPROVAL DECLARATION

Authors declare human ethics approval was not needed for this study.

## Supporting information


**Supplementary Figure 1.** A schematic protocol and timeline of the longitudinal prospective imaging study.

**Supplementary Figure 2.** Boxplots illustrating the (A) left and (B) right renal length, and (C) left and (D) right cortical thickness of the kidneys between normocalcemic and hypercalcemic CKD cats at baseline visit (termed baseline) and follow‐up visit (termed repeated).

**Supplementary Table 1.** Descriptive statistics on baseline ultrasonographical variables for CKD cats enrolled in this prospective imaging study, grouped according to the ionized calcium status at enrolment (“CKD normocalcemia” vs “CKD hypercalcemia”).

**Supplementary Table 2.** Linear mixed model and generalized linear mixed model analyses examining the change in clinicopathological variables over time in all enrolled CKD cats (n = 36) during the study period. Summary of intercepts and the slopes between groups (“absence of nephrocalcinosis” or “presence of nephrocalcinosis”).

**Supplementary Table 3.** A 2 × 2 paired sample contingency table illustrating the proportion of CKD cats with differing renal shape in left and right kidneys between baseline and repeated ultrasound scans.

**Supplementary Table 4.** A 2 × 2 paired sample contingency table illustrating the proportion of CKD cats with differing medullary rim sign in left and right kidneys between baseline and repeated ultrasound scans.

**Supplementary Table 5.** A 3 × 3 paired sample contingency table illustrating the proportion of CKD cats with differing classification of corticomedullary definition in left and right kidneys between baseline and repeated ultrasound scans.

**Supplementary Table 6.** A 3 × 3 paired sample contingency table illustrating the proportion of CKD cats with differing classification of nephrocalcinosis in left and right kidneys between baseline and repeated ultrasound scans.
