# Supplementary material for: Risk factors and implications associated with ultrasound‐diagnosed nephrocalcinosis in cats with chronic kidney disease
Source: J Vet Intern Med. 2024 Mar 4;38(3):1563–76. doi: 10.1111/jvim.17034 (PMC11099775; doi:10.1111/jvim.17034)

SUPPLEMENTARY MATERIAL

FIGURE 1. A schematic protocol and timeline of the longitudinal prospective imaging study.

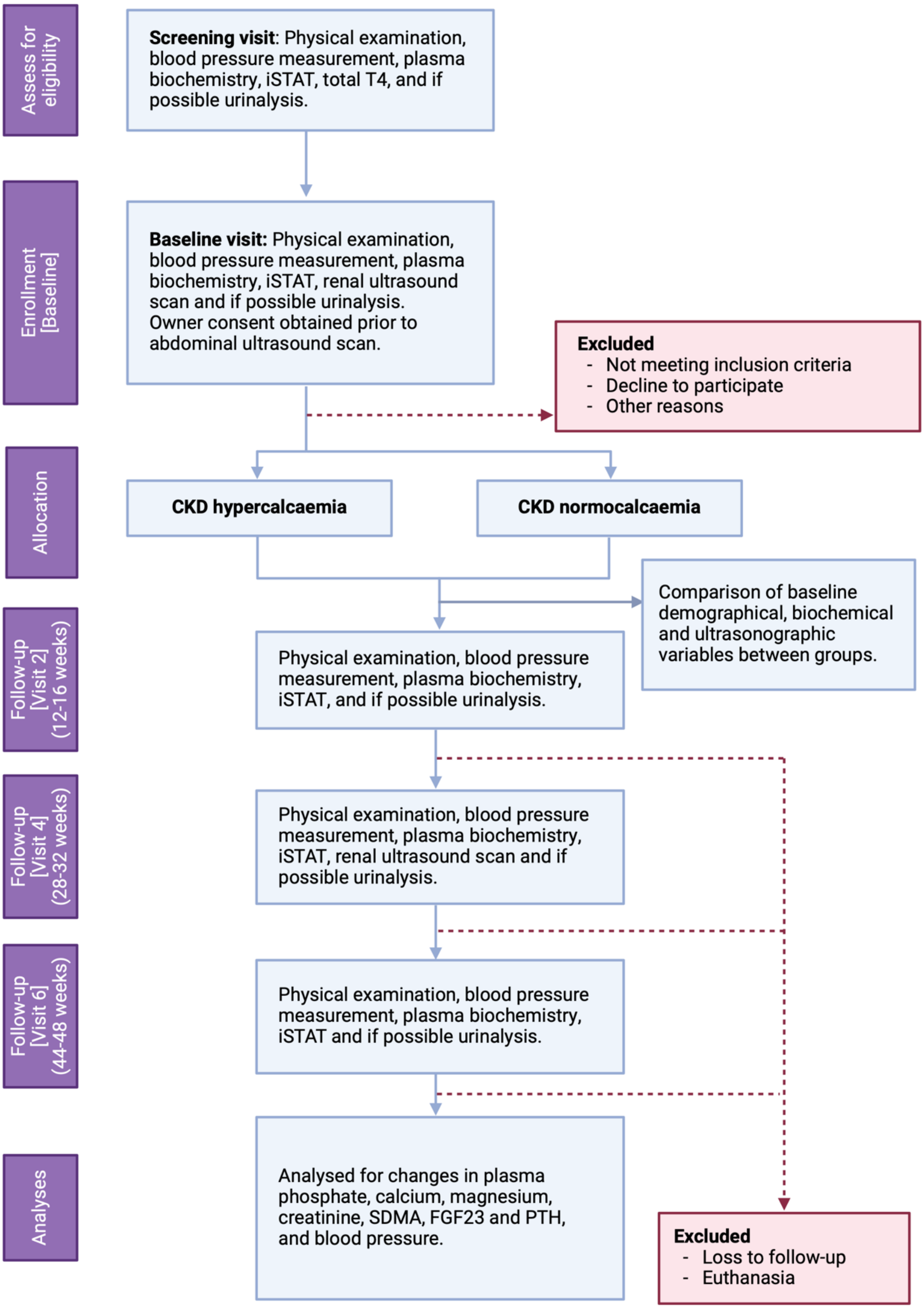

Supplement: Supplementary file 1 — Supplementary Figure 1. A schematic protocol and timeline of the longitudinal prospective imaging study. [file JVIM-38-1563-s003.pdf]
