# Supplementary material for: Risk factors and implications associated with ultrasound‐diagnosed nephrocalcinosis in cats with chronic kidney disease
Source: J Vet Intern Med. 2024 Mar 4;38(3):1563–76. doi: 10.1111/jvim.17034 (PMC11099775; doi:10.1111/jvim.17034)

SUPPLEMENTARY MATERIAL

FIGURE 2. Boxplots illustrating the (A) left and (B) right renal length, and (C) left and (D) right cortical thickness of the kidneys between normocalcemic and hypercalcemic CKD cats at baseline visit (termed baseline) and follow-up visit (termed repeated).

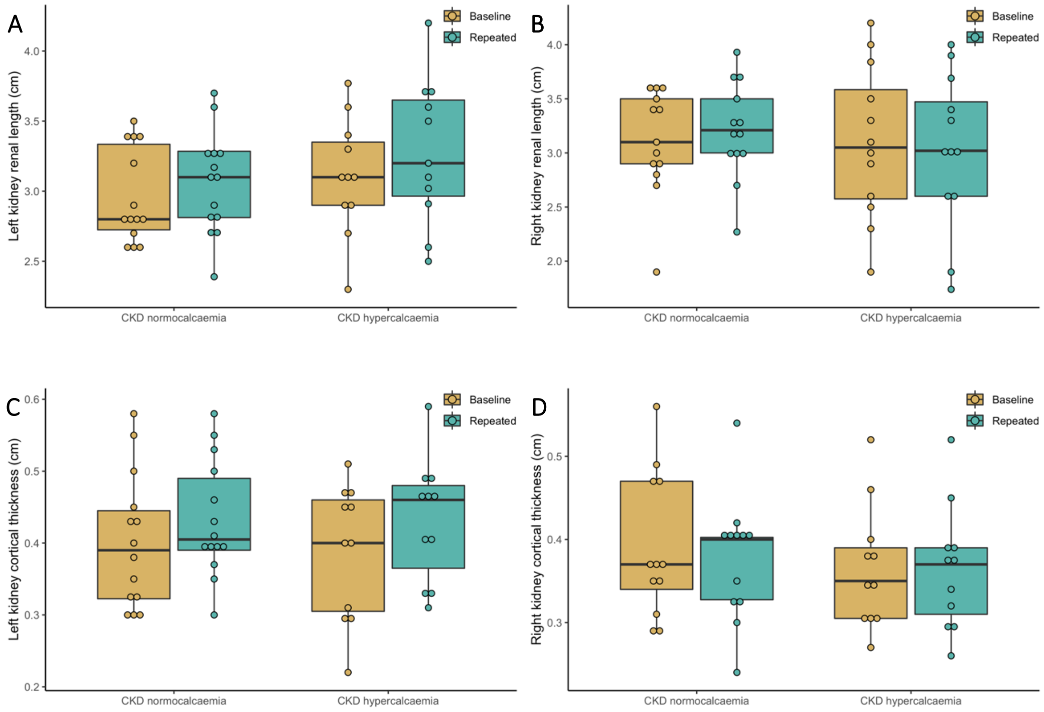

Supplement: Supplementary file 2 — Supplementary Figure 2. Boxplots illustrating the (A) left and (B) right renal length, and (C) left and (D) right cortical thickness of the kidneys between normocalcemic and hypercalcemic CKD cats at baseline visit (termed baseline) and follow‐up visit (termed repeated). [file JVIM-38-1563-s006.pdf]
