# Supplementary material for: Risk factors and implications associated with ultrasound‐diagnosed nephrocalcinosis in cats with chronic kidney disease
Source: J Vet Intern Med. 2024 Mar 4;38(3):1563–76. doi: 10.1111/jvim.17034 (PMC11099775; doi:10.1111/jvim.17034)
Supplement: Supplementary file 3 — Supplementary Table 1. Descriptive statistics on baseline ultrasonographical variables for CKD cats enrolled in this prospective imaging study, grouped according to the ionized calcium status at enrolment (“CKD normocalcemia” vs “CKD hypercalcemia”). [file JVIM-38-1563-s008.pdf]

SUPPLEMENTARY MATERIAL

TABLE 1. Descriptive statistics on baseline ultrasonographical variables for CKD cats enrolled in this prospective imaging study, grouped according to the ionized calcium status at enrolment (“CKD normocalcemia” vs. “CKD hypercalcemia”).

**Variables**	**CKD normocalcemia (n = 20)**		**CKD hypercalcemia (n = 16)**		*P*-value
	Median [25th, 75th Percentile]	n	Median [25th, 75th Percentile]	n	
Renal shape (left kidney)	Normal, 12; Irregular, 8	20	Normal, 11; Irregular, 5	16	.59
Renal shape (right kidney)	Normal, 12; Irregular, 8	20	Normal, 11; Irregular, 5	16	.59
Renal length (left kidney, cm)	3.0 [2.8, 3.4]	20	3.1 [3, 3.5]	15	.41
Renal length (right kidney, cm)	3.2 [2.9, 3.5]	20	3.1 [2.6, 3.6]	15	.68
Cortico-medullary differentiation (left kidney)	Maintained, 0; Poorly maintained, 11; Loss, 9	20	Maintained, 3; Poorly maintained, 6; Loss, 7	16	.13
Cortico-medullary differentiation (right kidney)	Maintained, 0; Poorly maintained, 12; Loss, 8	20	Maintained, 2; Poorly maintained, 9; Loss, 5	16	.36
Cortical thickness (left kidney, cm)	0.37 [0.32, 0.44]	20	0.4 [0.36, 0.47]	15	.4
Cortical thickness (right kidney, cm)	0.36 [0.31, 0.46]	18	0.37 [0.30, 0.40]	14	.99
Medullary rim sign (left kidney)	Present, 3 Absent, 17	20	Present, 3 Absent, 12	15	1
Medullary rim sign (right kidney)	Present, 3 Absent, 17	20	Present, 2 Absent, 13	15	1
Nephrocalcinosis (left kidney)	Present, 5; Suspected, 3; Absent, 12	20	Present, 5; Suspected, 5; Absent, 6	16	.35
Nephrocalcinosis (right kidney)	Present, 2; Suspected, 2; Absent, 16	20	Present, 1; Suspected, 7; Absent, 8	16	.06
Nephrolithiasis (left kidney)	Present, 1; Absent, 19	20	Present, 3; Absent, 12	15	.29
Nephrolithiasis (right kidney)	Present, 0; Absent, 20	20	Present, 1; Absent, 14	15	.43
Other parenchymal abnormality (left kidney)	Cortical scars, 2; Cortical cysts, 5; None, 13	20	Cortical scars, 1; Cortical cysts, 5; None, 9	15	.88
Other parenchymal abnormality (right kidney)	Cortical scars, 1; Cortical cysts, 1; None, 18	20	Cortical scars, 2; Cortical cysts, 3; None, 10	15	.26
Perirenal abnormality (left kidney)	None, 20	20	None, 15	15	1
Perirenal abnormality (right kidney)	None, 20	20	None, 15	15	1

Abbreviation: n, number of cats.
