# Supplementary material for: Risk factors and implications associated with ultrasound‐diagnosed nephrocalcinosis in cats with chronic kidney disease
Source: J Vet Intern Med. 2024 Mar 4;38(3):1563–76. doi: 10.1111/jvim.17034 (PMC11099775; doi:10.1111/jvim.17034)
Supplement: Supplementary file 4 — Supplementary Table 2. Linear mixed model and generalized linear mixed model analyses examining the change in clinicopathological variables over time in all enrolled CKD cats (n = 36) during the study period. Summary of intercepts and the slopes between groups (“absence of nephrocalcinosis” or “presence of nephrocalcinosis”). [file JVIM-38-1563-s005.pdf]

SUPPLEMENTARY MATERIAL

TABLE 2. Linear mixed model and generalized linear mixed model analyses examining the change in clinicopathological variables over time in in all enrolled CKD cats (n = 36) during the study period. Summary of intercepts and the slopes between groups (“absence of nephrocalcinosis” or “presence of nephrocalcinosis”).

**Variables**	**Absence of nephrocalcinosis (n = 14)**			**Presence of nephrocalcinosis (n = 22)**		
	Intercept	Coefficient of time (β; month)	*P*-value	Intercept	Coefficient of time (β; month)	*P*-value
BCS (“1–3”, “4–9”)	NA	(-0.18) ± 0.10	.06	NA	(-0.08) ± 0.08	.33
MCS (“0”, “1”, “2”, “3”)	NA	(-0.13) ± 0.07	.06	NA	(-0.06) ± 0.05	.29
Body weight (kg)	3.6 ± 0.2	(-0.01) ± 0.01	.32	3.9 ± 0.2	(-0.02) ± 0.01	**<.001**
Albumin (g/dL)	2.9 ± 0.1	0.011 ± 0.006	.06	3.1 ± 0.1	(-0.003) ± 0.004	.55
ALP (U/L)	28 ± 4	0.5 ± 0.3	.12	27 ± 4	(0.01) ± 0.22	.97
ALT (U/L)	41 ± 8	0.8 ± 0.8	.31	65 ± 6	0.2 ± 0.6	.76
Creatinine (mg/dL)	2.3 ± 0.3	0.02 ± 0.02	.2	2.9 ± 0.2	0.03 ± 0.01	**.04**
ln[FGF23] (pg/mL)	5.84 ± 0.35	(-0.035) ± 0.023	.14	6.53 ± 0.27	0.024 ± 0.017	.15
Venous HCO_3^- (mEq/L)	21.6 ± 0.7	0.01 ± 0.07	.86	21.0 ± 0.6	(-0.04) ± 0.06	.53
Ionized calcium (mg/dL)	5.40 ± 0.13	(-0.016) ± 0.014	.26	5.66 ± 0.10	(-0.011) ± 0.011	.32
PCV (%)	33 ± 1	0.24 ± 0.12	.06	31 ± 1	(-0.002) ± 0.095	.98
Venous pH	7.37 ± 0.01	(-0.001) ± 0.001	.69	7.35 ± 0.01	(-0.002) ± 0.001	.14
Phosphate (mg/dL)	3.8 ± 0.3	0.04 ± 0.02	.07	4.3 ± 0.2	0.06 ± 0.02	**<.001**
Potassium (mEq/L)	3.62 ± 0.12	0.012 ± 0.012	.32	3.87 ± 0.09	(-0.013) ± 0.010	.19

ln[PTH] (pg/mL)	2.18 ± 0.42	0.055 ± 0.035	.12	2.44 ± 0.32	(-0.032) ± 0.026	.22
SBP (mmHg)	131 ± 4	0.4 ± 0.4	.32	127 ± 3	(0.03) ± 0.30	.93
SDMA (µg/dL)	19 ± 2	0.1 ± 0.2	.58	23 ± 2	0.2 ± 0.1	.19
Sodium (mEq/L)	146 ± 2	0.5 ± 0.3	.13	151 ± 2	(-0.1) ± 0.2	.72
Total calcium (mg/dL)	9.99 ± 0.25	0.027 ± 0.019	.15	10.69 ± 0.20	0.007 ± 0.014	.63
Total magnesium (mg/dL)	2.02 ± 0.10	0.016 ± 0.012	.19	2.18 ± 0.08	0.005 ± 0.009	.6
Total protein (g/dL)	8.0 ± 0.2	0.003 ± 0.016	.83	7.6 ± 0.2	0.005 ± 0.012	.7

Outcome variables showing significant rate of change in each group (*P* ≤ .05) are highlighted in bold (gradient of regression line significantly different from 0). The unit used for time was month (30.4 days). Results are presented as coefficient (β) ± standard error.

Abbreviations: n, number of cats; BCS, body condition score; MCS, muscle condition score; ALP, alkaline phosphatase; ALT, alanine aminotransferase; HCO_3^-, bicarbonate; ln[FGF23], log-transformed fibroblast growth factor-23; OR, odd ratio; PCV, packed cell volume; ln[PTH], log-transformed parathyroid hormone; SBP, systolic blood pressure; SDMA, symmetric dimethylarginine
