# Supplementary material for: Risk factors and implications associated with ultrasound‐diagnosed nephrocalcinosis in cats with chronic kidney disease
Source: J Vet Intern Med. 2024 Mar 4;38(3):1563–76. doi: 10.1111/jvim.17034 (PMC11099775; doi:10.1111/jvim.17034)
Supplement: Supplementary file 5 — Supplementary Table 3. A 2 × 2 paired sample contingency table illustrating the proportion of CKD cats with differing renal shape in left and right kidneys between baseline and repeated ultrasound scans. [file JVIM-38-1563-s001.pdf]

SUPPLEMENTARY MATERIAL

TABLE 3. A 2 x 2 paired sample contingency table illustrating the proportion of CKD cats with differing renal shape in left and right kidneys between baseline and repeated ultrasound scans.

				Repeated		n	*P*-value
				Normal	Irregular		
**Renal shape (left)**	*Baseline*	All	Normal	14 (52%)	4 (15%)	27	.68
			Irregular	2 (7%)	7 (26%)		
		Normocalcemia	Normal	8 (57%)	1 (7%)	14	1
			Irregular	2 (14%)	3 (21%)		
		Hypercalcemia	Normal	6 (46%)	3 (23%)	13	.25
			Irregular	0 (0%)	4 (31%)		
**Renal shape (right)**	*Baseline*	All	Normal	17 (65%)	1 (4%)	26	.37
			Irregular	1 (4%)	4 (15%)		
		Normocalcemia	Normal	9 (69%)	0 (0%)	13	.48
			Irregular	2 (15%)	2 (15%)		
		Hypercalcemia	Normal	8 (62%)	1 (8%)	13	1
			Irregular	2 (15%)	2 (15%)		

Abbreviation: n, number of cats.
