# Supplementary material for: Risk factors and implications associated with ultrasound‐diagnosed nephrocalcinosis in cats with chronic kidney disease
Source: J Vet Intern Med. 2024 Mar 4;38(3):1563–76. doi: 10.1111/jvim.17034 (PMC11099775; doi:10.1111/jvim.17034)
Supplement: Supplementary file 6 — Supplementary Table 4. A 2 × 2 paired sample contingency table illustrating the proportion of CKD cats with differing medullary rim sign in left and right kidneys between baseline and repeated ultrasound scans. [file JVIM-38-1563-s002.pdf]

SUPPLEMENTARY MATERIAL

TABLE 4. A 2 x 2 paired sample contingency table illustrating the proportion of CKD cats with differing medullary rim sign in left and right kidneys between baseline and repeated ultrasound scans.

				Repeated		n	*P*-value
				Absent	Present		
**Medullary rim sign (left)**	*Baseline*	All	Absent	19 (73%)	3 (12%)	26	.68
			Present	1 (4%)	3 (12%)		
		Normocalcemia	Absent	9 (64%)	3 (21%)	14	1
			Present	1 (7%)	1 (7%)		
		Hypercalcemia	Absent	10 (83%)	0 (0%)	12	.25
			Present	0 (0%)	2 (17%)		
**Medullary rim sign (right)**	*Baseline*	All	Absent	18 (72%)	4 (16%)	25	.37
			Present	0 (0%)	3 (12%)		
		Normocalcemia	Absent	8 (62%)	3 (23%)	13	.48
			Present	0 (0%)	2 (15%)		
		Hypercalcemia	Absent	10 (83%)	1 (8%)	12	1
			Present	0 (0%)	1 (8%)		

Abbreviation: n, number of cats.
