# Supplementary material for: Risk factors and implications associated with ultrasound‐diagnosed nephrocalcinosis in cats with chronic kidney disease
Source: J Vet Intern Med. 2024 Mar 4;38(3):1563–76. doi: 10.1111/jvim.17034 (PMC11099775; doi:10.1111/jvim.17034)
Supplement: Supplementary file 7 — Supplementary Table 5. A 3 × 3 paired sample contingency table illustrating the proportion of CKD cats with differing classification of corticomedullary definition in left and right kidneys between baseline and repeated ultrasound scans. [file JVIM-38-1563-s004.pdf]

**SUPPLEMENTARY MATERIAL**

**TABLE 5.** A 3 x 3 paired sample contingency table illustrating the proportion of CKD cats with differing classification of cortico-medullary definition in left and right kidneys between baseline and repeated ultrasound scans.

| | | | | *Repeated* | | | n | *P*-value |
|---|---|---|---|---|---|---|---|---|
| | | | | Maintained | Poorly maintained | Loss | | |
| **Cortico-medullary definition (left)** | *Baseline* | All | Maintained | 1 (4%) | 1 (4%) | 0 (0%) | 27 | .11 |
| | | | Poorly maintained | 5 (19%) | 9 (33%) | 1 (4%) | | |
| | | | Loss | 0 (0%) | 2 (7%) | 8 (30%) | | |
| | | Normocalcemia | Maintained | 0 (0%) | 0 (0%) | 0 (0%) | 14 | .23 |
| | | | Poorly maintained | 3 (21%) | 5 (36%) | 1 (7%) | | |
| | | | Loss | 0 (0%) | 1 (7%) | 4 (29%) | | |
| | | Hypercalcemia | Maintained | 1 (8%) | 1 (8%) | 0 (0%) | 13 | .42 |
| | | | Poorly maintained | 2 (15%) | 4 (31%) | 0 (0%) | | |
| | | | Loss | 0 (0%) | 1 (8%) | 4 (31%) | | |
| **Cortico-medullary definition (right)** | *Baseline* | All | Maintained | 0 (0%) | 2 (8%) | 0 (0%) | 26 | .43 |
| | | | Poorly maintained | 5 (19%) | 8 (31%) | 3 (12%) | | |
| | | | Loss | 0 (0%) | 3 (12%) | 5 (19%) | | |
| | | Normocalcemia | Maintained | 0 (0%) | 0 (0%) | 0 (0%) | 13 | .07 |
| | | | Poorly maintained | 3 (23%) | 6 (46%) | 0 (0%) | | |
| | | | Loss | 0 (0%) | 1 (8%) | 3 (23%) | | |
| | | Hypercalcemia | Maintained | 0 (0%) | 2 (15%) | 0 (0%) | 13 | .79 |
| | | | Poorly maintained | 2 (15%) | 2 (15%) | 3 (23%) | | |
| | | | Loss | 0 (0%) | 2 (15%) | 2 (15%) | | |

Abbreviation: n, number of cats.
