# Supplementary material for: Risk factors and implications associated with ultrasound‐diagnosed nephrocalcinosis in cats with chronic kidney disease
Source: J Vet Intern Med. 2024 Mar 4;38(3):1563–76. doi: 10.1111/jvim.17034 (PMC11099775; doi:10.1111/jvim.17034)
Supplement: Supplementary file 8 — Supplementary Table 6. A 3 × 3 paired sample contingency table illustrating the proportion of CKD cats with differing classification of nephrocalcinosis in left and right kidneys between baseline and repeated ultrasound scans. [file JVIM-38-1563-s007.pdf]

SUPPLEMENTARY MATERIAL

TABLE 6. A 3 x 3 paired sample contingency table illustrating the proportion of CKD cats with differing classification of nephrocalcinosis in left and right kidneys between baseline and repeated ultrasound scans.

				Repeated			n	*P*-value
				Absent	Present (suspected)	Present		
			Absent	11 (41%)	2 (7%)	0 (0%)		
		All	Present (suspected)	0 (0%)	3 (11%)	3 (11%)	27	.59
			Present	1 (4%)	1 (4%)	6		
			Absent	8 (57%)	1 (7%)	0 (0%)		
**Nephrocalcinosis (left)**	*Baseline*	Normocalcemia	Present (suspected)	0 (0%)	1 (7%)	1 (7%)	14	1
			Present	1 (7%)	0 (0%)	2 (14%)		
			Absent	3 (23%)	1 (8%)	0 (0%)		
		Hypercalcemia	Present (suspected)	0 (0%)	2 (15%)	2 (15%)	13	.42
			Present	0 (0%)	1 (8%)	4 (31%)		
			Absent	13 (50%)	4 (15%)	1 (4%)		
		All	Present (suspected)	1 (4%)	3 (12%)	2 (8%)	26	.1
			Present	0 (0%)	1 (4%)	1 (4%)		
			Absent	9 (69%)	1 (8%)	1 (8%)		
**Nephrocalcinosis (right)**	*Baseline*	Normocalcemia	Present (suspected)	0 (0%)	1 (8%)	0 (0%)	13	.37
			Present	0 (0%)	0 (0%)	1 (8%)		
			Absent	4 (31%)	3 (23%)	0 (0%)		
		Hypercalcemia	Present (suspected)	1 (8%)	2 (15%)	2 (15%)	13	.3
			Present	0 (0%)	1 (8%)	0 (0%)		

Abbreviation: n, number of cats.
